# Amelioration of Chromium VI Toxicity in Sorghum (*Sorghum bicolor* L.) using Glycine Betaine

**DOI:** 10.1038/s41598-019-52479-w

**Published:** 2019-11-05

**Authors:** Praveen Kumar, Jayanti Tokas, H. R. Singal

**Affiliations:** Department of Biochemistry, College of Basic Sciences and Humanities, CCSHAU Hisar, 125004 Haryana India

**Keywords:** Chemical modification, Oxidoreductases, Metabolomics, Abiotic, Climate-change mitigation

## Abstract

The main objective of the present research work was to study the effect of Cr toxicity and its amelioration by glycine betaine (GB) in sorghum (HJ 541 and SSG 59-3). Chromium (Cr VI), 2 and 4 ppm led to a significant reduction in plant height, root length, chlorophyll content, antioxidant enzymes viz. catalase, peroxidase, ascorbate peroxidase, glutathione reductase, polyphenol oxidase, and superoxide dismutase; and metabolites viz. ascorbate, proline, and glutathione. The results of the present study supported the findings that the application of GB can minimize or reduce the toxic effects caused by Cr VI which reaches the plants via soil, water, and air pollution. It is concluded that GB at both 50, as well as 100 mM concentrations, successfully ameliorated Cr VI (up to 4 ppm) toxicity and its application may be recommended for crops affected by Cr VI toxicity to get better growth and yield.

## Introduction

Sorghum (*Sorghum bicolor* L.) is a versatile crop of Poaceae family grown for food, fodder, and industrial revolutions. Sorghum is a C4 plant that usually grows in a hot and dry environment. Globally, sorghum is cultivated in 42.12 million hectares, and India ranks second in terms of area under sorghum cultivation. The production is estimated to be 61.38 million tons globally and 5.28 million tons in India^1^. The sorghum cultivation area in the state of Haryana covered approx. 76 thousand hectares on an average during 2013–18 and production was around 43 ton bales^−1^ ^2^. About 300 million people depends on this cereal grass for their nutritional requirements.

However, environmental changes cause great losses to agricultural production in the world^3^. The growth and production of sorghum are usually affected by different abiotic stresses like drought, salinity, temperature, and heavy metal (HM) toxicity. Among HM toxicities, chromium VI toxicity has established harmful effects on a living system^4^. The main source of Cr (VI) pollution is tanning industries. Low- and middle-income countries contribute towards major part of world’s tanning industry and their contribution has increased from 35% to 56% and 26% to 56%, respectively between 1970 and 2010^5^. Many of these tannery sites are clustered together, creating heavily polluting industrial areas in these countries. As per Blacksmith’s inventory of sites, South Asia (India and Pakistan particularly) has the highest number of tanning industries, with South America also at risk of the large population being exposed to Cr contamination^6^. Major Cr contaminated cities in India include Ranipet, Kanpur, Vadodara, and Talcher^7^. In Haryana, plant growth of field crops has been affected in industrial areas like Sonepat, Dharuhera, Shahbad, Faridabad, Gurgaon, Yamunanagar, Karnal, Panchkula, and Panipat^8^.

Chromium toxicity value ranges from 21 to 47 ppm in Haryana, whereas the permissible value is 0.05 to 0.5 ppm both in water and soil^9^. It exists in soil mainly in two forms; trivalent and hexavalent depending on its oxidation state. The hexavalent form is more toxic compared to the trivalent form because the former has greater solubility than later. However, both the forms are interconvertible depending on the oxidation-reduction reaction occurring in the soil elements. The modern industrial activities like manufacturing of refractory steel, boring muds, coating of washing agents, catalytic creation, tanning of leather as well as manufacturing of chromic acid are the common sources of hexavalent Cr in the environment. In plants, it causes reduced photosynthesis, free radical generation, inhibition of plant growth, wilting of tops, chlorosis of young leaves, destruction of roots and finally death of the plant. When exposed to different stresses, certain metabolic shifts occur in plants resulting in varied levels of cellular metabolites^10^. These cellular modifications, in response to abiotic stress, may appear to be associated with the enhanced ability of the plants to tolerate such conditions.

Many organisms have developed a common strategy of accumulation of compatible solutes/osmolytes, to overcome the environmental stresses^11,12^. Among these, the most common are betaines, polyols, polyamines, sugars (mannitol, sorbitol, and trehalose), and amino acid (proline), which play a protective role for plants under abiotic stresses. Glycine betaine (GB) is one of the major organic solute that gets accumulated in a number of plant species in response to different environmental stresses. GB is expected to contribute to enhancing the HM stress tolerance in plants. The tolerant or sensitive species may be differentiated depending on the accumulated amount of GB during heavy metal chromium stress. GB is non-toxic, soluble in water^13^ and one of the best-studied compatible solutes^14^. It is a quaternary ammonium compound that is found in bacteria, marine invertebrates, hemophilic archaebacteria, plants and mammals^15,16^. It gets accumulated to significant levels in salt-tolerant plants and halotolerant cyanobacteria^17,18^. The GB level varies significantly among different plant species and organs. Low levels of GB are found to be in the plants of distant species (taxonomically). However, when plants are subjected to abiotic stresses^19^, large amounts of GB accumulation has been reported. However, there are some plant species which do not produce GB under normal or stressful conditions^19^.

The available literature indicated that GB plays an important role in the amelioration of heavy metal Cr (VI) toxicity by increasing the activity of the antioxidative enzymes of the plant. Keeping the above view, the present study was planned to examine the effect of Cr (VI) toxicity and GB application on different morphophysiological & biochemical parameters in sorghum plants. Two different sorghum cultivars were selected for this experimental study, on the basis that SSG 59-3 is a multicut^20^ while HJ 541 is a single cut cultivar. Moreover, SSG 59-3 is sweeter than HJ 541. They are widely grown in Haryana region for the nourishment of animals and industrial purposes. They are the only source of forage in dryland during the summer season. Both the cultivars differ from each other in quality parameters. However, there are no reports about the sensitivity of two cultivars against Cr (VI) stress.

## Results

GB ameliorated the toxic effect of Cr (VI) stress on plant growth, chlorophyll content, antioxidative enzymes, and metabolites reflecting a significant increase in their amounts. The physical appearance of sorghum plants differ significantly in control plants, GB treated plants and Cr (VI) stressed plants. The plants with Cr (VI) treatment alone were shorter than those grown with GB (50 & 100 mM) treatments. The results of the present study are as follows.

### Effect of exogenous GB on Cr (VI) induced suppression in Morphophysiological parameters

**Figure 1:**
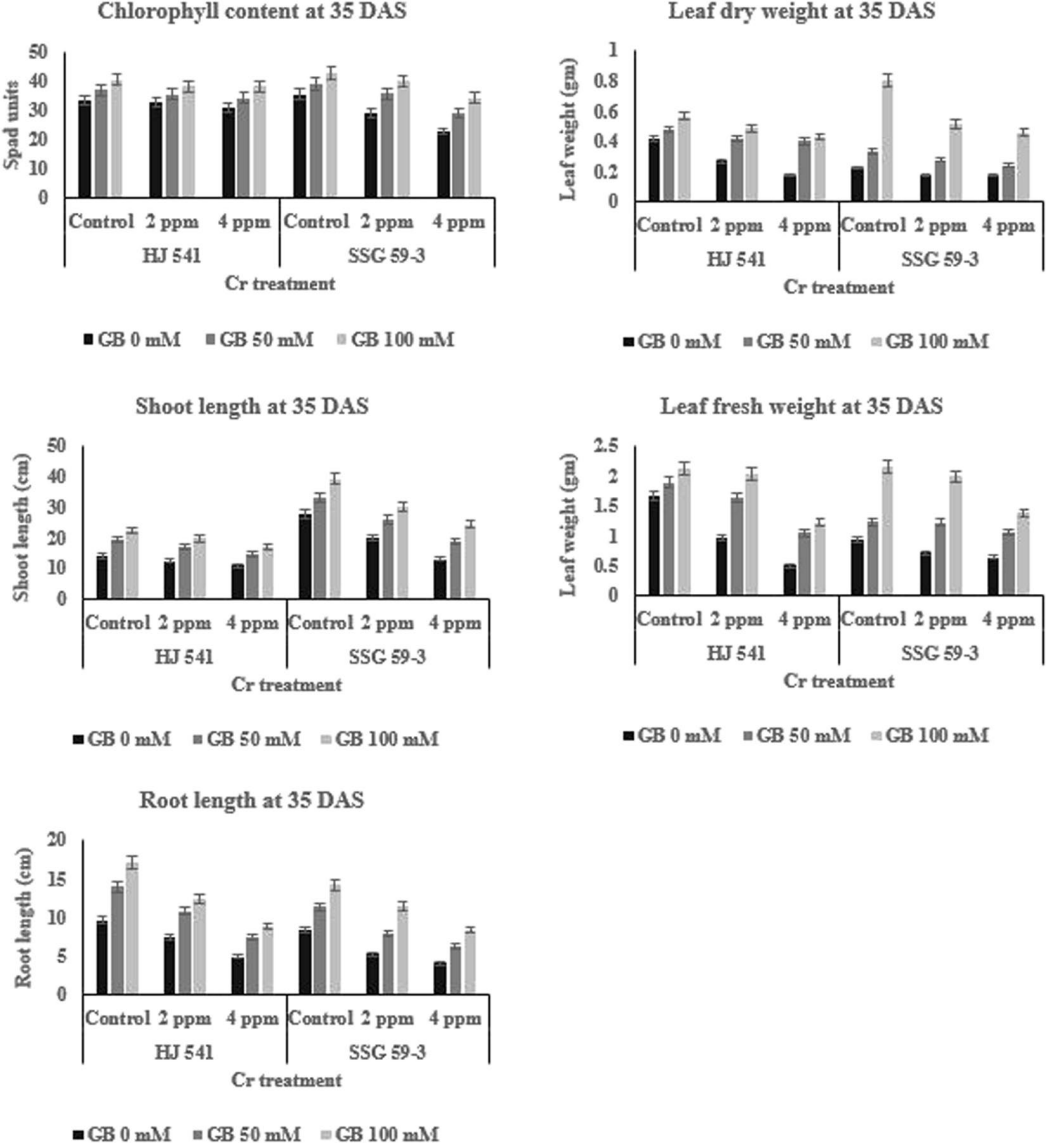
Effects of various Glycine betaine treatments on morphophysiological parameters like chlorophyll content, leaf dry weight, shoot length, leaf fresh weight and root length at 35 days after sowing (DAS) growth stage of sorghum plants grown under Cr stress. Values represent the mean ± S.E. from three independent experiments. Significance difference was at P ≤ 0.05 (ANOVA).

**Figure 2:**
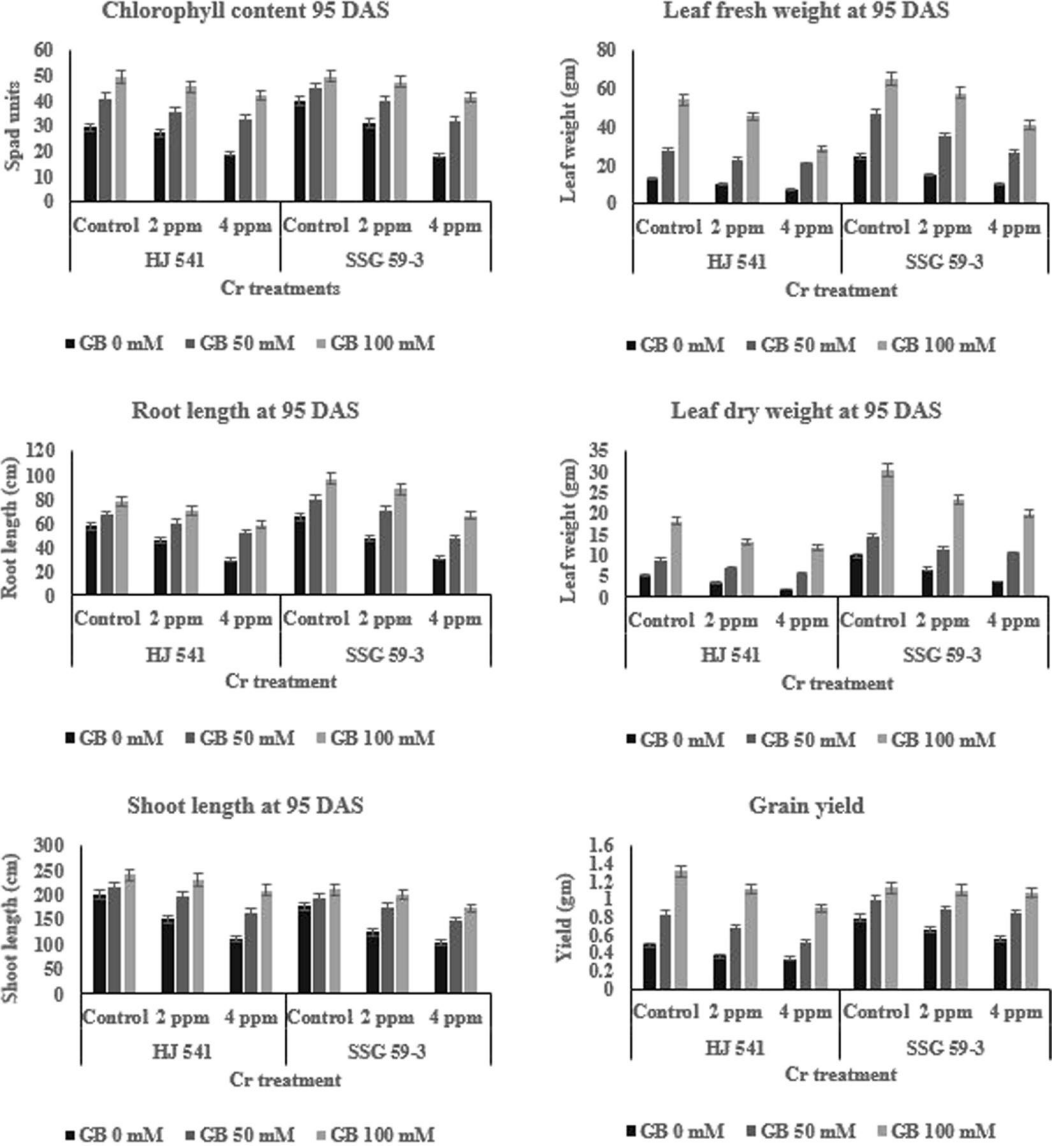
Effects of various Glycine betaine treatments on morphophysiological parameters like chlorophyll content, leaf fresh weight, root length, leaf dry weight, shoot length and grain yield at 95 DAS growth stage of sorghum plants grown under Cr stress. Values represent the mean ± S.E. from three independent experiments. Significance difference was at P ≤ 0.05 (ANOVA).

Chromium VI toxicity reduced the plant growth and development with increasing Cr concentrations (0–4 ppm). The effect of Cr (VI) toxicity on growth and development of sorghum was evaluated by six characters, i.e. fresh weight, dry weight, root length, shoot length, chlorophyll content and grain yield. The effect of exogenously supplied GB on growth characters of Sorghum plants under Cr (VI) stress is shown in Figs 1 and 2. The results obtained show that the growth characters of sorghum plants significantly decreased under chromium stress in comparison with control plants.

#### Chlorophyll content

There was a decrease of 0.77% and 6.71% at 35 DAS (Fig. 1) and 7.43% and 35.44% at 95 DAS (Fig. 2) of chlorophyll content in HJ 541 plants grown under 2 and 4 ppm Cr, respectively. In SSG 59–3, the decrease of chlorophyll content was 18.32% and 35.75% at 35 DAS and 22.43% and 54.61% at 95 DAS under 2 and 4 ppm Cr, respectively. The results showed that GB at both concentrations (50 and 100 mM) significantly increased the chlorophyll content in both the varieties (Figs 1 and 2). The increase in chlorophyll content was 4.42% and 7.81% in HJ 541 and 8.57% and 25.94% in SSG 59-3 plants grown under 2 ppm and 4 ppm Cr, respectively; at 35 DAS on 50 mM GB application. The increase at 95 DAS was 12.16% and 19.98% in HJ 541 and 10.91% and 28.73% in SSG 59-3 plants grown under 2 ppm and 4 ppm Cr, respectively; by 50 mM GB treatment. The treatment, 100 mM of GB further increased the chlorophyll content by 5.41% and 5.32% in HJ 541 and 6.56% and 20.10% in SSG 59-3 plants grown under 2 ppm and 4 ppm Cr, respectively; at 35 DAS. The increase at 95 DAS was 7.84% and 15.04% in HJ 541 and 4.70% and 17.15% in SSG 59-3 plants grown under 2 ppm and 4 ppm Cr, respectively; by 100 mM GB treatment.

#### Dry weight content

It was observed that leaf dry weight decreased by 35.35% and 58.11% at 35 DAS (Fig. 1) and 31.78% and 64.59% at 95 DAS (Fig. 2) in HJ 541 plants grown under 2 and 4 ppm Cr, respectively. In SSG 59-3, the decrease of leaf dry weight was 20.63% and 22.42% at 35 DAS and 31.74% and 62.68% at 95 DAS under 2 and 4 ppm Cr, respectively. The results showed that GB at both concentrations (50 and 100 mM) significantly increased leaf dry weight in both the varieties (Figs 1 and 2). The increase in leaf dry weight was 12.58% and 16.14% in HJ 541 and 16.82% and 27.93% in SSG 59-3 plants grown under 2 and 4 ppm Cr, respectively; at 35 DAS on 50 mM GB application. The increase at 95 DAS was 21.34% and 37.33% in HJ 541 and 19.13% and 28.20% in SSG 59-3 plants grown under 2 and 4 ppm Cr, respectively; by 50 mM GB treatment. The 100 mM GB, treatment further increases leaf dry weight by 14.11% and 24.16% in HJ 541 and 35.88% and 42.88% in SSG 59-3 plants grown under 2 and 4 ppm Cr, respectively; at 35 DAS. The increase in leaf dry weight at 95 DAS was 28.38% and 34.55% in HJ 541 and 23.62% and 34.24% in SSG 59-3 plants grown under 2 and 4 ppm Cr, respectively; by 100 mM GB treatment.

#### Fresh weight content

The leaf fresh weight decreased by 41.92% and 70.66% at 35 DAS (Fig. 1) and 25.70% and 43.64% at 95 DAS (Fig. 2) in HJ 541 plants grown under 2 and 4 ppm Cr; respectively. The decrease for leaf fresh weight content in SSG 59-3 was 23.91% and 32.76% at 35 DAS and 38.72% and 59.54% at 95 DAS under 2 and 4 ppm Cr, respectively. The results showed that GB at both concentrations (50 and 100 mM) significantly increased leaf fresh weight content in both the varieties (Figs 1 and 2). The increase in leaf fresh weight content was 13.60% and 44.61% in HJ 541 and 1.38% and 14.88% in SSG 59-3 plants grown under 2 and 4 ppm Cr, respectively; at 35 DAS, on 50 mM GB application. The increase at 95 DAS was 18.82% and 26.98% in HJ 541 and 24.85% and 43.67% in SSG 59-3 plants grown under 2 and 4 ppm Cr, respectively; by 50 mM GB treatment. The 100 mM GB treatment further increased leaf fresh weight by 4.68% and 43.24% in HJ 541 and 7.31% and 35.86% in SSG 59-3 plants grown under 2 and 4 ppm Cr, respectively; at 35 DAS. The increase in leaf fresh weight at 95 DAS was 16.34% and 47.31% in HJ 541 and 12.08% and 37.42% in SSG 59-3 plants grown under 2 and 4 ppm Cr, respectively; by 100 mM GB treatment.

#### Shoot length

There was decrease 11.20% and 22.53% at 35 DAS (Fig. 1) and 24.58% and 44.85% at 95 DAS (Fig. 2) in HJ 541 plants grown under 2 and 4 ppm Cr, respectively. In SSG 59-3, the decrease in shoot length was 27.27% and 53.47% at 35 DAS and 29.60% and 40.89% at 95 DAS under 2 and 4 ppm Cr, respectively. The results showed that GB in both concentrations (50 and 100 mM) significantly increased shoot length in both the varieties (Figs 1 and 2). The increase in shoot length was 11.39% and 23.92% in HJ 541 and 21.16% and 43.40% in SSG 59-3 plants grown under 2 and 4 ppm Cr, respectively; at 35 DAS on 50 mM GB application. The increase in shoot length at 95 DAS was 10.51% and 22.47% in HJ 541 and 11.71% and 39.90% in SSG 59-3 plants grown under 2 and 4 ppm Cr, respectively; on 50 mM GB application. The treatment of 100 mM GB further increased the shoot length by 11.38% and 23.80% in HJ 541 and 23.09% and 38.14% in SSG 59-3 plants grown under 2 and 4 ppm Cr, respectively; at 35 DAS. The increase in shoot length at 95 DAS was 9.90% and 25.15% in HJ 541 and 8.85% and 31.83% in SSG 59-3 plants grown under 2 and 4 ppm Cr, respectively; by 100 mM GB treatment.

#### Root length

The root length decreased by 22.53% and 49.31% at 35 DAS (Fig. 1) and 20.81% and 48.34% at 95 DAS (Fig. 2) in HJ 541 plants grown under 2 and 4 ppm Cr, respectively. In SSG 59-3, the root length decreased by 37.56% and 51.71% at 35 DAS and 27.53% and 52.64% at 95 DAS under 2 and 4 ppm Cr, respectively. The results showed that GB in both concentrations (50 and 100 mM) significantly increased root length in both the varieties (Figs 1 and 2). The increase in root length was 22.86% and 47.50% in HJ 541 and 29.94% and 45.07% in SSG 59-3 plants grown under 2 and 4 ppm Cr, respectively; at 35 DAS, on the application of 50 mM GB. The increase in root length was 10.51% and 22.47% in HJ 541 and 11.71% and 39.90% in SSG 59-3 plants grown under 2 and 4 ppm Cr, respectively; at 95 DAS, by 50 mM GB treatment. On the increase of GB concentration up to 100 mM, root length was increased further by 27.41% and 48.32% in HJ 541 and 20.02% and 41.47% in SSG 59-3 plants grown under 2 and 4 ppm Cr, respectively; at 35 DAS. The increase in root length at 95 DAS was 9.90% and 25.15% in HJ 541 and 8.85% and 31.83% in SSG 59-3 plants grown under 2 and 4 ppm Cr, respectively; at the same concentration, 100 mM GB treatment.

#### Grain yield

It was observed that there was 24.95% and 31.64% decrease in grain yield of HJ 541 under 2 and 4 ppm Cr, respectively. In SSG 59-3, the decrease of grain yield was 15.51% and 29.76% under 2 and 4 ppm Cr, respectively; (Fig. 2). The exogenous application of GB (50 and 100 mM) significantly increased grain yield in both the varieties (Fig. 2). The increase in grain yield was 17.23% and 36.99% in HJ 541 and 10.81% and 15.15% in SSG 59-3 plants grown under 2 and 4 ppm Cr, respectively; by 50 mM GB treatment. The yield was increased further by 15.49% and 31.13% in HJ 541 and 2.04% and 4.42% in SSG 59-3 plants grown under 2 and 4 ppm Cr, respectively; on 100 mM GB application.

It owes, therefore, be concluded that both 50 and 100 mM concentrations of GB significantly improved the growth characters against chromium toxicity in sorghum.

### Effect of Cr (VI) and exogenous GB on Antioxidative defense system of sorghum plants

#### Effect on antioxidative enzyme activities

**Figure 3:**
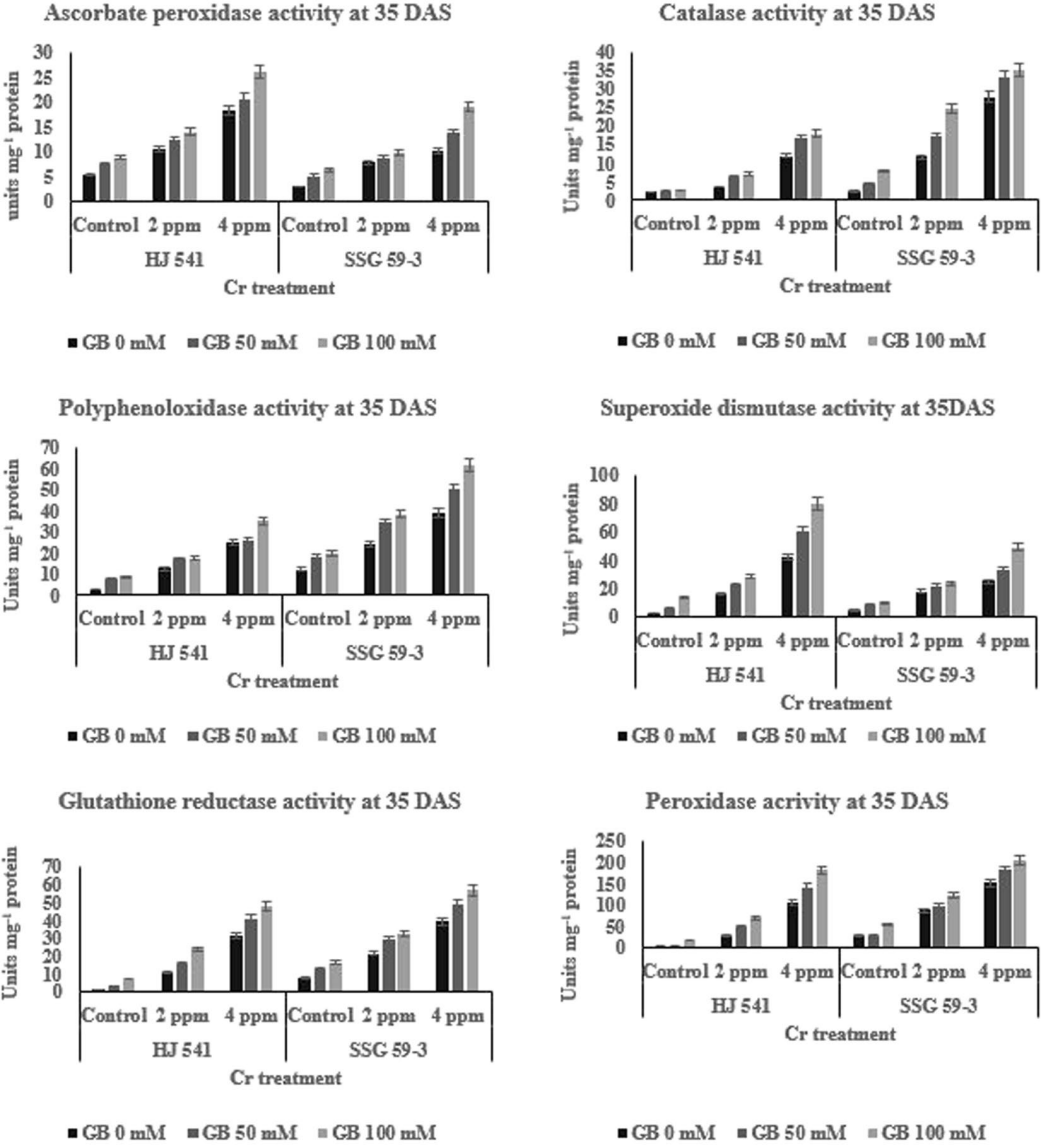
Changes in the activities of various Antioxidative enzymes (ascorbate peroxidase, catalase, polyphenol oxidase, superoxide dismutase, glutathione reductase and peroxidase) of sorghum grown under Cr toxicity, on various levels of GB application at 35 DAS growth stage. Values represent the mean ± S.E. from three independent experiments. Significance difference was at P ≤ 0.05 (ANOVA).

**Figure 4:**
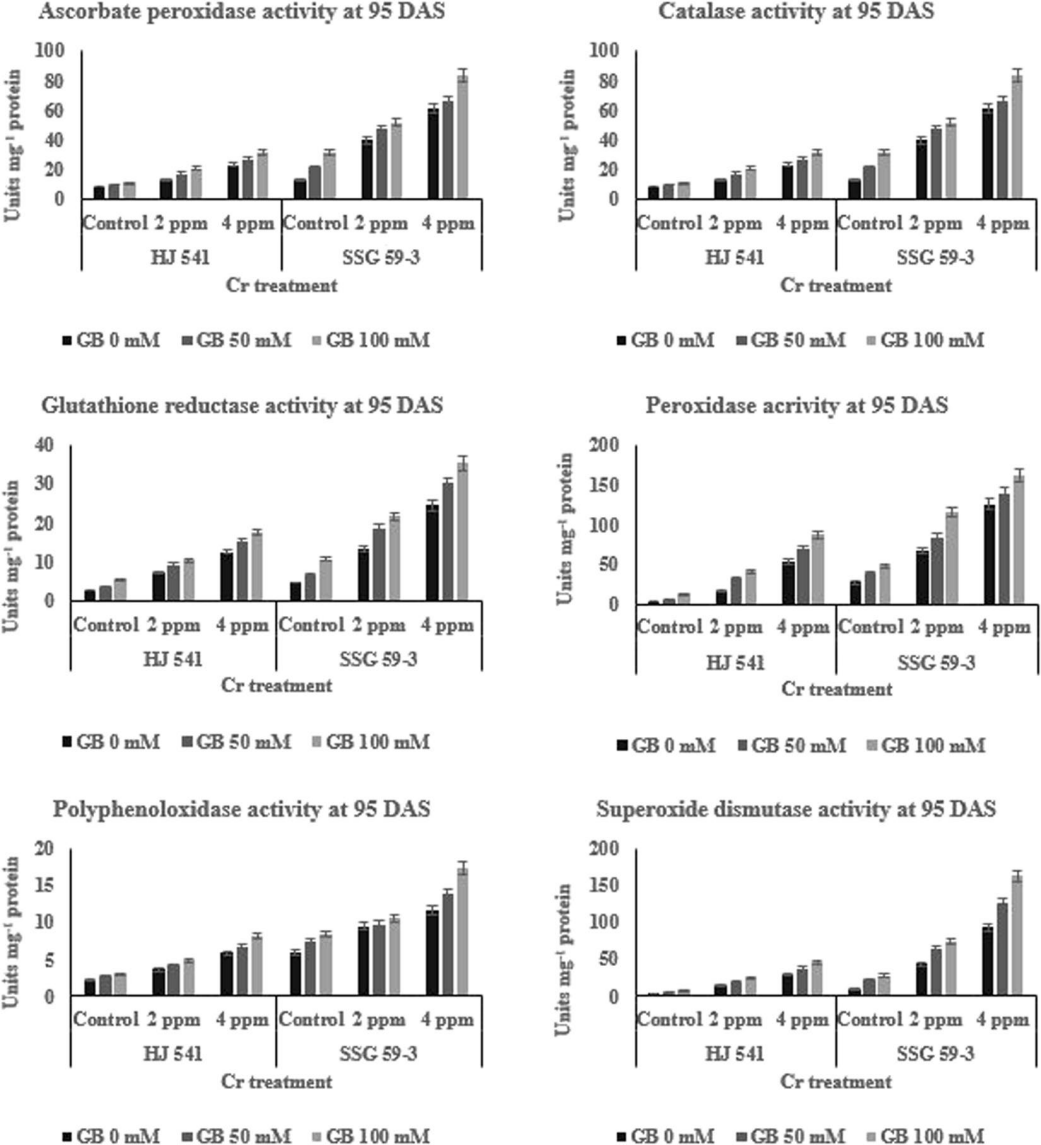
Changes in the activities of various Antioxidative enzymes (ascorbate peroxidase, catalase, glutathione reductase, peroxidase, polyphenoloxidase and superoxide dismutase) of sorghum grown under Cr toxicity, on various levels of GB application at 95 DAS growth stage. Values represent the mean ± S.E. from three independent experiments. Significance difference was at P ≤ 0.05 (ANOVA).

The results showed that the activity of all the enzymes (Figs 3 and 4), viz. ascorbate peroxidase, catalase, polyphenol oxidase, superoxide dismutase, glutathione reductase, and peroxidase increased (46–49%) with the increasing concentration of chromium (2 & 4 ppm) as compared to control at both stages (35 & 95 DAS) and in both the varieties. GB treatment at both concentrations (50 & 100 mM) further increased (29–32%) the activity of all the enzymes as compared to Cr treated plants alone, in both the varieties at both stages. The enzyme activity of ascorbate peroxidase was high in variety HJ 541 compared to SSG 59-3. But, the values of all the remaining enzyme activities were more in SSG 59-3 variety compared to HJ 541, which indicated that the former can tolerate the toxic stress, especially chromium toxicity, more strongly.

#### Effect on antioxidative metabolites level

**Figure 5:**
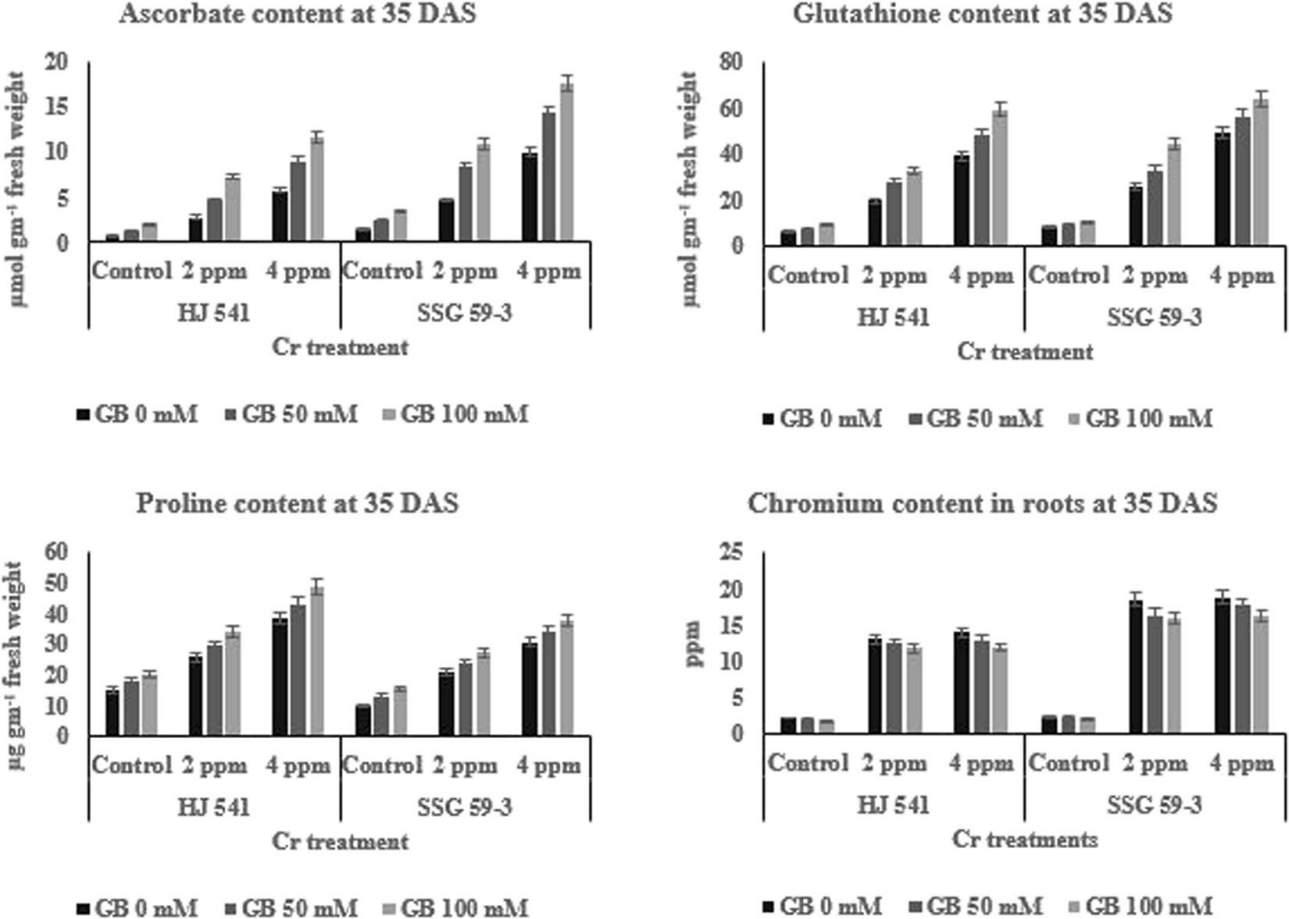
Effects of GB treatments on various Antioxidative metabolites (ascorbate, glutathione, proline) and chromium accumulation in sorghum plants grown under Cr toxic stress at 35 DAS growth stage. Values represent the mean ± S.E. from three independent experiments. Significance difference was at P ≤ 0.05 (ANOVA).

**Figure 6:**
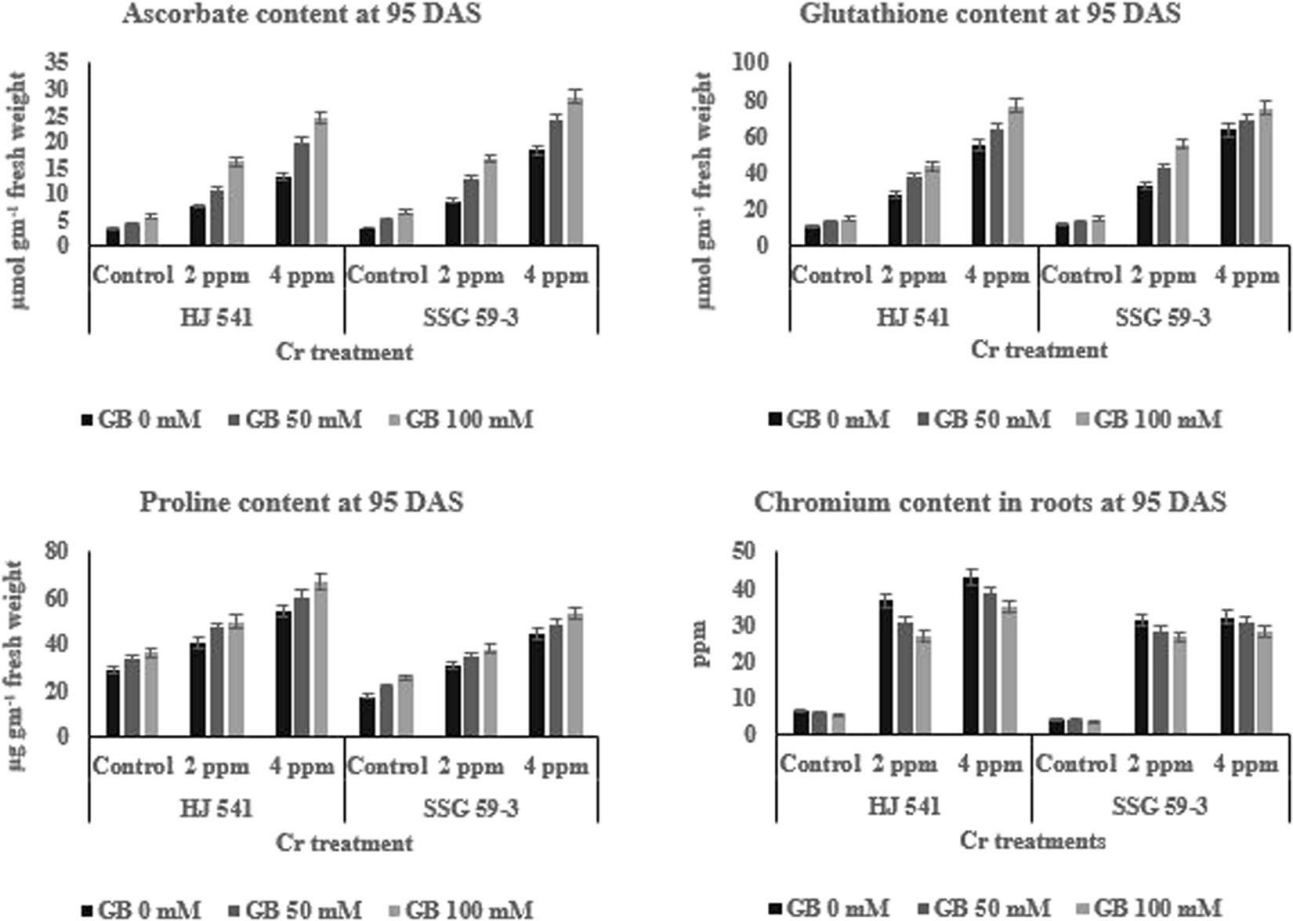
Effects of GB treatments on various Antioxidative metabolites (ascorbate, glutathione, proline) and chromium accumulation in sorghum plants grown under Cr toxic stress at 95 DAS growth stage. Values represent the mean ± S.E. from three independent experiments. Significance difference was at P ≤ 0.05 (ANOVA).

The results (Figs 3 and 4) indicated the same pattern for antioxidative metabolites as for antioxidative enzymes during Cr (VI) stress at both the stages in both varieties. The content of all metabolites viz. ascorbate, glutathione, and proline increased with increasing concentrations of Cr VI and was highest at 4 ppm. However, the treatment of GB at 50 mM further increased the content of by 12.38% for ascorbate, 9.41% for glutathione and 4.36% for proline, significantly at 35 DAS in HJ 541 plants grown under 2 ppm Cr (Fig. 3). At 4 ppm Cr, the increase was 12.46% for ascorbate, 6.48% for glutathione and 3.63% for proline at 35 DAS in HJ 541 plants. The increase in antioxidative metabolites was highest 15.25% for ascorbate, 7.21% for glutathione and 4% for proline at 100 mM concentration of GB at 35 DAS in both varieties (Figs 5 and 6). The proline content was observed more in HJ 541 compared to SSG 59-3 at both stages (35 & 95 DAS). But, the ascorbate and glutathione content was almost similar in both the varieties at both stages. These findings again favor the stronger tolerance nature of HJ 541 than SSG 59-3 variety.

### Effect of exogenous GB on Cr-VI accumulation in sorghum plant

It was observed that the Cr (VI) content in the sorghum roots increased significantly (3–4%) with the increase in Cr (VI) supply at both stages in both varieties. The content of Cr (VI) in roots of sorghum also increased along with the growth stage (35 to 95 DAS) of the plant in both varieties (Figs 5 and 6). The exogenous application of GB at both (50 & 100 mM) concentrations caused significant reduction (5–8%) in the absorption of Cr (VI) from soil to the sorghum roots in both varieties at both stages. These results suggested that the Cr (VI) toxicity was significantly reduced by the application of GB in sorghum plants grown under chromium stress.

### Effect of exogenous GB on Forage quality parameters (ADF, NDF, cellulose, hemicellulose, lignin, pectin, and silica) of sorghum plants under Cr-VI stress

Acid detergent fiber (ADF), Neutral detergent fiber (NDF), cellulose, hemicellulose lignin, pectin, and silica are less digestible by digestive system of animals and acts as indications for quality of forage crop for animal nutrition. In the present study, it was observed that the content of all these parameters increased (50–60%) with increasing concentration of Cr (VI) as compared to control, at both stages in both varieties (Tables 1 and 2), respectively. But the treatment of GB at 50 and 100 mM concentrations decreased the content (23–33%) of all these parameters at both Cr, levels (2 & 4 ppm) in both varieties at both stages. The decrease was more at 100 mM treatment of GB under 2 ppm Cr stress as compared to 4 ppm of Cr toxic stress and 50 mM GB treatment in both varieties at both stages. It was observed that GB 50 and 100 mM caused a significant reduction in ADF, NDF, cellulose, hemicellulose, lignin, pectin, and silica content at both toxic levels of Cr in both the varieties at both the stages.

**Table 1:** Effect of Glycine betaine treatments on the level of various quality metabolites (ADF, NDF, Cellulose, Hemicellulose, Lignin, Pectin and Silica) in sorghum plants under Cr VI stress as compared to control plants at 35 DAS growth stage.

Variety	Treatments	ADF^a^	NDF^a^	Cellulose^a^	Hemicellulose^a^	Lignin^a^	Pectin^b^	Silica^a^
HJ 541	Control	16.23	22.61	17.86	6.48	11.11	2.81	6.59
	50 mM GB	14.01	19.39	14.42	5.41	8.55	2.53	5.54
	100 mM GB	13.00	16.39	11.43	3.51	5.43	2.04	3.33
	2 ppm Cr	41.92	64.17	47.91	23.18	33.62	4.35	15.97
	2 ppm Cr + 50 mM GB	33.98	52.33	37.05	19.75	27.92	3.90	13.69
	2 ppm Cr + 100 mM GB	26.92	41.51	26.77	14.69	22.49	3.36	9.73
	4 ppm Cr	48.56	80.59	64.81	31.42	44.38	5.13	22.14
	4 ppm Cr + 50 mM GB	40.36	70.54	55.27	30.26	39.28	4.72	20.11
	4 ppm Cr + 100 mM GB	32.67	60.28	42.44	26.57	34.15	4.29	17.81
SSG 59-3	Control	19.08	22.99	12.39	4.32	5.59	1.91	4.36
	50 mM GB	18.42	21.28	10.50	3.17	4.90	1.79	3.10
	100 mM GB	18.01	20.59	8.95	2.60	3.47	1.64	2.42
	2 ppm Cr	39.55	60.36	35.88	20.54	30.38	3.03	10.40
	2 ppm Cr + 50 mM GB	32.52	49.39	26.61	17.34	25.72	2.68	8.64
	2 ppm Cr + 100 mM GB	26.99	38.31	19.51	11.02	17.93	2.32	6.77
	4 ppm Cr	44.62	71.46	49.41	26.67	41.58	3.68	16.99
	4 ppm Cr + 50 mM GB	38.54	62.54	41.64	24.24	36.58	3.39	15.00
	4 ppm Cr + 100 mM GB	31.99	50.98	32.37	18.88	32.87	2.98	12.98
**CD Values**		**ADF**	**NDF**	**Cellulose**	**Hemicellulose**	**Lignin**	**Pectin**	**Silica**
**P-** ^**Value≤0.05**^	Factor (A)	NS	0.582	0.433	0.158	0.259	0.034	0.117
	Factor (B)	0.368	0.713	0.530	0.194	0.318	0.041	0.143
	Factor (C)	0.368	0.713	0.530	0.194	0.318	0.041	0.143
	Intraction (A × B)	0.521	1.009	0.75	0.274	0.449	0.058	0.202
	Intraction (A × C)	0.521	NS	0.75	0.274	0.449	0.058	0.202
	Intraction (B × C)	0.638	1.235	0.919	0.336	0.550	0.071	0.248
	Intraction (A × B × C)	NS	NS	NS	0.475	0.778	0.101	0.350

^a^Each values are expressed as % dry weight bases of sample;

^b^Each values are in µg/gm dry weight bases;

Factor (A): Variety;

Factor (B): Cr (VI) treatment;

Factor (C): GB treatment;

Values represent the mean ± S.E. from three independent experiments; significance difference at P ≤ 0.05 (ANOVA).

CD values signifies significant effects of respective treatments. NS stands for non-significant effect of respective treatment.

**Table 2:** Effect of Glycine betaine treatment on the level of various quality metabolites in sorghum plants under Cr VI stress as compared to control plants at 95 DAS growth stage.

Variety	Treatments	ADF^a^	NDF^a^	Cellulose^a^	Hemicellulose^a^	Lignin^a^	Pectin^b^	Silica^a^
HJ 541	Control	28.76	34.46	25.74	5.65	25.48	7.24	12.38
	50 mM GB	26.47	29.79	23.16	4.32	22.27	6.40	10.37
	100 mM GB	24.99	28.58	20.69	3.61	18.51	5.58	8.31
	2 ppm Cr	50.99	75.28	56.17	24.50	47.24	9.01	20.97
	2 ppm Cr + 50 mM GB	41.57	61.77	46.42	20.40	39.19	8.24	18.74
	2 ppm Cr + 100 mM GB	34.33	48.76	35.45	13.74	32.01	7.05	15.96
	4 ppm Cr	61.23	87.85	73.45	27.96	59.58	11.02	27.61
	4 ppm Cr + 50 mM GB	50.43	73.28	62.40	24.50	55.58	10.02	24.61
	4 ppm Cr + 100 mM GB	45.34	63.04	50.13	18.21	49.77	9.00	22.54
SSG 59-3	Control	23.70	25.41	22.77	2.38	16.83	4.03	9.61
	50 mM GB	21.94	22.86	20.28	2.20	14.00	3.78	7.54
	100 mM GB	19.58	22.16	18.45	2.01	11.73	3.42	5.49
	2 ppm Cr	49.38	68.07	53.21	17.15	41.34	6.48	17.82
	2 ppm Cr + 50 mM GB	38.46	57.49	44.86	12.18	35.01	5.90	15.09
	2 ppm Cr + 100 mM GB	31.42	36.93	34.66	5.58	28.09	5.07	12.00
	4 ppm Cr	56.05	80.62	64.66	24.48	53.56	7.76	23.62
	4 ppm Cr + 50 mM GB	47.07	69.39	54.38	22.06	48.01	7.14	20.17
	4 ppm Cr + 100 mM GB	39.53	51.47	44.43	19.13	41.64	6.09	17.03
**CD Values**		**ADF**	**NDF**	**Cellulose**	**Hemicellulose**	**Lignin**	**Pectin**	**Silica**
**P-** ^**Value**≤ **0.05**^	Factor (A)	0.381	0.582	0.433	0.158	0.259	0.034	0.117
	Factor (B)	0.467	0.713	0.530	0.194	0.318	0.041	0.143
	Factor (C)	0.467	0.713	0.530	0.194	0.318	0.041	0.143
	Intraction (A × B)	0.660	1.009	0.750	0.274	0.449	0.058	0.202
	Intraction (A × C)	NS	NS	0.750	0.274	0.449	0.058	0.202
	Intraction (B × C)	0.809	1.235	0.919	0.336	0.550	0.071	0.248
	Intraction (A × B × C)	NS	NS	NS	0.475	0.778	0.101	0.350

^a^Each values are expressed as % dry weight bases of sample;

^b^Each values are in µg/gm dry weight bases;

Factor (A): Variety;

Factor (B): Cr VI treatment

Factor (C): GB treatment;

Values represent the mean ± S.E. from three independent experiments; significance difference at P ≤ 0.05 (ANOVA).

CD values signifies significant effects of respective treatments. NS stands for non-significant effect of respective treatment.

The content of ADF, NDF, cellulose, hemicellulose, lignin, pectin, and silica also increased (40–45%) significantly along with growth stages of the plant in both varieties. But, the increase in quality parameters was more in HJ 541 compared to SSG 59–3. Moreover, the later was found to be more tolerant towards Cr toxicity in comparison to the former at both stages. The high rate of decrease in the content of these parameters was observed at 100 mM concentration of GB at both stages in both varieties.

## Discussion

Chromium toxicity has become a serious problem in agricultural soil all over the world and requires an immediate solution^21,22^. Chromium (VI) pollution has produced many negative effects on plant’s and animal’s health^23^. High concentrations of Cr (VI) inhibit seed germination and plant growth by affecting many biochemical and physiological processes such as protein synthesis, photosynthesis, enzymatic and non-enzymatic antioxidative defense system (viz. catalase, peroxidase, superoxide dismutase, ascorbate peroxidase, glutathione reductase, polyphenol oxidase, and metabolites glutathione, proline, and ascorbate)^24,25^. Chromium (VI) toxicity also affects the quality and resistance capacity of plants^26,27^. In the present research, efforts have been made to study the ameliorative role of exogenously supplied GB in sorghum plants grown under different concentrations (2 & 4 ppm) of Cr (VI). The findings of this research work are in agreement with various other researchers reported in other species till now^28,29^.

### Exogenous GB reduces Cr (VI) accumulation in sorghum plants and counteracts nutrients elements changes

During the present study, it was observed that Cr (VI) levels increased in various plant parts with increase in Cr (VI) levels (Figs 5 and 6) in the soil as compared to control plants. Similar observations were also made by other researchers in moong bean plants grown under Cr stress^30–32^. It might be due to the change in EC, pH and OC properties (Table 3) of the soil on Cr (VI) application. It is well reported by Gomes et al^33^. during his study on the absorption of Cr, Cd, Cu, Ni, Zn and Pb by the plants. Soil properties (pH, EC, OC) has a significant effect on the sorption of HM in soils^34^. A low pH value leads to a reduction in sorption which consequently enhances the bioavailability or mobility of HM^35^. The presence of organic matter in the soil has a major influence on the nature of trace metals like Cr. Organic matter possess negatively charged surfaces which play a significant role in cation exchange capacity in the soil^36^. It causes more availability of positively charged metals like Cr to plant roots and results in increased Cr level in Cr (VI) treated plants. GB application in soil decreased the Cr accumulation and total Cr uptake by sorghum plants compared to respective Cr (VI) treatment alone. The reduction in uptake of heavy metal like Cd and Pb by plant roots because of GB application was also reported earlier in mung bean, rice, and cotton crops^37–39^. It might be due to the shielding nature of GB that inhibits the entry of Cr (VI) in the cytoplasm via cell membrane or the other way of competition between Cr (VI) with other nutrients’ uptake by the plant^40^.

**Table 3:** Initial properties of the soil used for the research work.

Property	Value and unit (2016–17)	Values and units (2017–18)	Evaluation
pH	8.2	7.9	Basic
Organic carbon (OC)	0.32	0.37	Low
Electrical conductivity (EC)	0.17 DS meter^−1^	0.19 DS meter^−1^	Normal
Nitrogen (N)	3 mg kg^−1^ soil	3.6 mg kg^−1^ soil	Low
Phosphorus (P)	8 mg kg^−1^ soil	7.8 mg kg^−1^ soil	Low
Potassium (K)	84 mg kg^−1^ soil	81 mg kg^−1^ soil	Normal
Zink (Zn)	0.61 mg kg^−1^ soil	0.63 mg kg^−1^ soil	Normal
Iron (Fe)	0.7 mg kg^−1^ soil	0.68 mg kg^−1^ soil	Low
Copper (Cu)	0.18 mg kg^−1^ soil	0.17 mg kg^−1^ soil	Normal
Manganese (Mn)	2.73 mg kg^−1^ soil	3 mg kg^−1^ soil	Normal
Chromium (Cr)	0.016 mg kg^−1^ soil	0.014 mg kg^−1^ soil	Low

### Exogenous GB offsets Cr (VI) induced inhibition in Morphophysiological Parameters

The results of the present study have shown (Figs 1 and 2) that 4 ppm chromium greatly reduced the chlorophyll content in sorghum plants. But, the application of GB (50 and 100 mM) significantly increased (25–27%) chlorophyll content. The maximum increase was observed in 100 mM treatment of GB in sorghum plants. GB application clearly affected the photosynthetic pigments and improved it, by increasing the plant performance like nutrient uptake and antioxidative defense system. Similar results were observed by Bharwana *et al*.^39^ in cotton crop under heavy metal lead (Pb) toxicity. The scientist observed that GB ameliorated Pb toxicity in cotton plants by inducing tolerance and elevating photosynthesis along with other responsible characters. Application of GB (50 & 100 mM) significantly increased (35–40%) the plant growth (root-shoot length & plant biomass) of sorghum under Cr (VI) toxicity compared to control plants (Figs 1 and 2). Similar observations have been made by Ali *et al*.^41^. The reason might be chelating nature of GB for Cr which blocks the movement of Cr from soil to plant and in plant parts. It reduces the Cr stress level in plants which in turn increased plant growth. The increased plant growth by GB, under Cr (VI) stress, might be due to the better development in nutrient uptake and gas exchange attributes of plants on GB application, as reported by Iqbal *et al*.^42^ and Shahbaz *et al*.^43^ in case of wheat under abiotic drought stress conditions. Moreover, GB may protect CO_2_ fixing enzymes like RuBisCo and RuBisCo activase under abiotic stress, and thus, leading to an improvement in plant growth^44^.

### Exogenous GB counteracts Cr-VI induced alterations in the Antioxidative defense system

Plants are able to protect themselves from the harmful effects of heavy metal stress by reducing reactive oxygen species (ROS) accumulation using enzymes, such as ascorbate peroxidase, catalase, superoxide dismutase, polyphenol oxidase, peroxidase, glutathione reductase and metabolites like glutathione, proline, and ascorbate^45,46^. The results of the present investigation (Figs 2–6) showed that GB application (50 & 100 mM) increased the activities of antioxidant enzymes and metabolite in Sorghum plants grown under chromium stress. Glycine betaine treatment significantly increased (25–28%) peroxidase and catalase enzymes activities compared to control as well as Cr (VI) treated plants. Reports suggested that ascorbate and proline may consume the ROS generated in plants due to stress conditions^47,48^. Proline, a basic amino acid, is found in high percentage in protein. Free proline plays a crucial role in plants during stress. Though the molecular mechanism has not yet been recognized regarding the increased level of proline, one of the hypotheses refers to the breakdown of protein into amino acids followed by conversion to proline for storage. Many researchers have reported a several-fold increase in the proline content under physiological and pathological stress conditions. Increased levels of glutathione, proline, and ascorbate with increasing concentration of GB under different treatments of Cr (VI) have also been observed in the present study (Figs 5 and 6) suggesting the protective role of GB against HM stress. Similar observations were also reported by Arafa *et al*.^49^ in sorghum plants under saline stress and Ali *et al*.^23^ in wheat under Cr stress. The GB treatments were found to be effective in the amelioration of Cr (VI) toxicity as evident from the better growth of sorghum plants (Figs 1 and 2) and reduction of Cr (VI) level in roots (Figs 5 and 6). Similar results were also obtained in case of rice and mung bean plants under Cadmium (Cd) stress^37,38^ and cotton under lead (Pb) stress with the exogenous application of GB^39^. Cha-um *et al*.^50^ reported the similar results in the activities of antioxidative enzymes by GB under drought stress. Park *et al*.^51^ reported that in tomato under chilling stress, the expression of catalase synthesis initiating genes was enhanced by GB application. The reason behind, the increase in the enzymatic activities after GB treatment might be due to the decrease in Cr uptake or reduction in electrolyte leakage^52^. The action of the antioxidative defense system (enzymatic and non-enzymatic) may protect the plant cells from oxidative damage by quenching or converting the ROS into harmless forms^53^.

In the present study, the activity of all antioxidative enzymes increased by the application of GB during the Cr stress. Similar observations were also reported by Gill *et al*.^53^ in their study on *Brassica napus* under Cr stress. The results of the present study also revealed an increase in antioxidative enzymes and metabolites activity in plants under Cr VI treatment alone (Figs 3 and 4). But, the plant growth was less in these plants (under 2 & 4 ppm of Cr treatment alone) as compared to control and plants provided with GB (Figs 1 and 2), which indicates that the increment in antioxidative enzyme activities under Cr treatment alone was not enough to support the plant growth and development compared to both control as well as GB treated plants. Moreover, continuous Cr stress leads to a reduction in the capacity of the antioxidative defense mechanism of sorghum plants against Cr stress. The decrease in activities of the antioxidative defense system in Cr treated plants causes’ reduction in the efficiency of antioxidants to consume ROS that increased the chances of ROS accumulation in the plant cell, which ultimately causes plant death. But the soil application of GB further increased the activity and efficiency of the antioxidative defense system in those plants (under 2 & 4 ppm of Cr treatment alone), which leads to the decreased stressed condition due to excess ROS, and increased growth and quality parameters by the reduction in Cr accumulation. It suggested the amelioration property of GB relating to Cr toxicity. These results were in accordance with Raza *et al*.^54^ and Molla *et al*.^55^ who studied the physiology of wheat and lentil under drought stress. They reported that the GB application mitigated the adverse effects of drought and improved the plant’s tolerance capacity to stress. Likewise, Einset *et al*.^56^ reported that GB might activate the expression of genes responsible for ROS scavenging enzyme synthesis, which may protect the photosynthetic apparatus of plants under stressful conditions. In the present study, increase in plant height, root length, chlorophyll content, and quality parameters might be due to the GB induced decrease in Cr uptake in plants and increase in activity of antioxidative enzymes as well as metabolites. GB increases or favors the growth of mycorrhizal fungi around plant roots. Mycorrhizal fungi reduce the HMs uptake by plant roots either by chelating HMs or storing more HMs in their vacuoles.

### Exogenous GB mitigates Cr (VI) induced damage to Forage Quality of sorghum

Forage quality parameters like ADF, NDF, cellulose, hemicellulose, lignin, pectin, and silica are the measures, which reveal the digestibility, i.e. how easily or in how much amount will an animal digest the feed. In other words, these parameters are used to determine the nutrition value of a particular crop for animal feed. The results of the present study (Tables 1 and 2) showed that chromium toxicity reduced the quality of sorghum by increasing the amount of ADF, NDF, cellulose, hemicellulose, lignin, pectin, and silica, but GB application increased the quality of sorghum by reducing the Cr absorption in sorghum roots (Figs 5 and 6) along with other processes like increased activity of antioxidative enzymes (Figs 3 and 4) and metabolites (Figs 5 and 6). Daud *et al*.^57^ observed that the plant cells under stressful conditions induce more lignification, silicification, ADF, NDF synthesis, to make the cell-wall stronger and thicker against osmotic burst. This favors the survival of plants by protecting the cells from osmotic stress caused by heavy metal. These results (Tables 1 and 2) were also in accordance with results obtained by Daud *et al*.^57^ in cluster beans. Thus, due to the decrease in Cr (VI) absorption by sorghum plants, on GB application the toxic stress was reduced in sorghum plants, which in turn induces plant cell to bring normal synthesis of lignification, silicification, and structural carbohydrates in the cell-wall that leads to enhanced quality of sorghum digestibility by the animals. This might be the reason for increased forage quality of sorghum on the GB application. Available reports in literature on Cr toxicity and tolerance reported that GB helps in chelation of heavy metals in the cellular vacuoles and causes the blockage of heavy metal movement or transportation^44^. This might be the reason behind the Cr VI toxicity tolerance and amelioration of toxic effects caused in sorghum by GB application which were recorded during this experimental study.

## Conclusion

From the results of the present investigation, it may be concluded that Cr (VI) is a non-essential element for plants and toxic heavy metal for sorghum that affects the plant morpho-physiological, biochemical quality at the molecular level. Application of exogenous GB has been found to inhibit Cr (VI) uptake by sorghum plants which might be due to GB induced chelation of heavy metal in cellular vacuoles. Thus, GB causes blockage of heavy metal Cr (VI) movement. This might be the reason behind the ameliorative effect of GB in sorghum also. Hence, application of exogenous GB may be used in the improvement of quality and yield of sorghum in Cr affected areas.

## Methods

The experiment was conducted in the Department of Biochemistry, CCS Haryana Agricultural University Hisar, India, during the years 2016–17 and 2017–18.

### Experimental design

Seeds of Sorghum variety HJ 541 and SSG 59-3 were procured from Forage Section, Department of GPB, CCS HAU, Hisar. The plants were raised in earthen pots. The pots were filled with 5 kg sandy loam soil and placed in a naturally lit screen house. The initial composition of the soil is given in Table 3.

### Treatments

Chromium VI treatments (2 & 4 ppm) were provided in the soil before sowing in the form of K_2_Cr_2_O_7_.7H_2_O. GB (50 & 100 mM) treatments were provided in the soil before sowing by using Betaine, B2629 from SIGMA. The treatment levels were maintained by analyzing the potting soil at different time intervals. Separate pots were maintained for the control group. All pots were irrigated with equal quantities of water and nutrient solution as per the package of practices (POP).

### Raising of the crop

The healthy seeds were selected and surface sterilized before sowing. Ten seeds per pot were sown at a depth of 5 cm. After seedling emergence, thinning was done up to six seedlings per pot.

### Sampling

The plant samples were collected and analyzed at 35 & 95 days after sowing (DAS) from each treatment. A complete plant was picked separately from each replication. A total of three replicates were prepared. The root, shoot, and leaves of the plant were collected separately for respective analysis.

### Morpho-physiological parameters

Among the morphological parameters, root length and shoot length were measured in cm by using a non-commercial scale. The biomass (fresh and dry weight) of the plant was measured in gm. The dry weight was determined in the same plant sample by keeping it in a hot air oven at 70 °C till the constant weight was achieved. The chlorophyll content was estimated using the Chlorophyll Meter, SPAD-502 Plus (Konica Minolta, Inc.). The grain yield was determined on 100 grains weight basis (100 grains were selected randomly and weighed).

### Biochemical parameters

Among biochemical parameters, structural carbohydrates, viz. neutral detergent fiber (NDF), acid detergent fiber (ADF), cellulose, hemicellulose, lignin, and silica were estimated by using the method of Van Soest and Wine^58^. Nonstructural carbohydrates viz. total sugar content was determined by using Dubois *et al*.’s^59^ method. Crude protein was estimated by implementing conventional Micro-Kjeldahl method (984.13) of AOAC^60^. Proline content in the straw sample was analyzed by applying the method of Bates *et al*.^61^ Chromium content in roots was estimated by the method of Sahuquillo *et al*.^62^ and expressed in ppm.

### Antioxidative system

The complete extraction procedure for both, the enzymes as well as metabolites was carried out, at 0–4 °C. Two gm of fresh and cleaned leaf tissue were homogenized in 10 ml of 0.1 M potassium phosphate buffer (pH 7.0) by using a previously chilled mortar and pestle. The homogenate was centrifuged at 10,000 rpm for 15 minutes. The supernatant labeled as crude extract was collected and used at the same time for measurement activity of all the enzymes as well as estimation of metabolites. The same crude extract was used for total soluble protein estimation.

#### Superoxide dismutase (EC 1.15.1.1)

Superoxide dismutase was assayed by measuring its ability to inhibit the photochemical reduction of nitro blue tetrazolium by adopting the method of Giannopolities and Ries^63^. One enzyme unit is defined as the amount of enzyme, which could cause 50% inhibition of the photochemical reaction.

#### Catalase

Catalase activity was determined by the procedure of Sinha^64^. One enzyme unit is defined as the amount of enzyme, which catalyzed the oxidation reaction of 1 µmole H_2_O_2_ minute^−1^ under assay conditions.

#### Peroxidase (EC 1.11.1.7)

The enzyme activity was estimated by the method of Shannon et al.^65^. One unit of peroxidase is defined as the amount of enzyme required to cause a change in 0.1 O.D. minute^−1^ under assay conditions.

#### Ascorbate peroxidase (EC 1.11.1.11)

The enzyme activity was determined by following the oxidation of ascorbic acid^66^. One enzyme unit is defined as the amount of enzyme required to oxidize 1 nmol of ascorbic acid minute^−1^ at 290 nm.

#### Glutathione reductase (EC 1.6.4.2)

Method of Halliwell and Foyer^67^ was followed to measure the enzyme activity. One enzyme unit is defined as the amount of enzyme required to oxidize 1.0 nmol of NADPH oxidized minute^−1^.

#### Polyphenol oxidase (E.C. 1.10.3.1)

Polyphenol oxidase activity was assayed by using the modified method of Taneja and Sachar^68^. One unit of enzyme activity is expressed as a change in 0.01 absorbance minute^−1^ mg^−1^ protein.

#### Ascorbate content

Ascorbic acid is an important antioxidant, when present in reduced form. It is widely distributed in fresh fruits like guava, mango, ber, papaya, and leafy vegetables such as cabbage and spinach. Ascorbic acid was determined by the slightly modified procedure of Oser^69^, which was originally developed by Roe^70^. The amount of ascorbate was determined by using a reference curve of ascorbate and expressed as µmoles gm^−1^ fresh weight.

#### Proline content

The estimation of the proline content in plants was examined by implementing the method of Bates *et al*.^61^. The amount of proline content present in the samples was determined from the standard curve of proline and has been expressed as µmoles gm^−1^ fresh weight.

#### Glutathione content

It is a major water-soluble antioxidant involved in maintaining the low redox potential and a highly reduced intracellular environment. It is also involved in scavenging of reactive oxygen species. Level of glutathione was estimated by using the method of Smith^71^. Glutathione content was calculated from a standard curve of GSH and is expressed as µmoles gm^−1^ fresh weight.

### Statistical analysis

All the results were analyzed by following a three-factorial (the First factor included varieties “2 varieties as HJ 541 and SSG 59-3”, second included chromium levels as control, 2 and 4 ppm, and the third factor included glycine betaine administration at control, 50 & 100 mM) analysis of variance (ANOVA) by using IBM SPSS Statistics 23 software along with post hoc Tukey test. On the basis of CD values obtained after this analysis for each parameter at both 35 and 95 DAS, differences between the treatment doses were evaluated^72^. Based on the ANOVA test, the interactions were found to be significant.

## Data Availability

All data generated or analyzed during this study are included in this article file.
